# Midterm outcomes of primary reverse shoulder arthroplasty: a systematic review of studies with minimum 5-year follow-up

**DOI:** 10.1016/j.xrrt.2023.09.003

**Published:** 2023-10-03

**Authors:** Tom R. Doyle, Sophia Downey, Eoghan T. Hurley, Christopher Klifto, Hannan Mullett, Patrick J. Denard, Grant E. Garrigues, Mariano E. Menendez

**Affiliations:** a Sports Surgery Clinic, Dublin, Ireland; b Department of Surgery, University of Galway, Galway, Ireland; c Royal College of Surgeons in Ireland, Dublin, Ireland; d Department of Orthopaedics, Duke University, Durham, NC, USA; e Oregon Shoulder Institute, Medford, OR, USA; f Department of Orthopaedics, Rush University, Chicago, IL, USA

**Keywords:** Shoulder arthroplasty, Reverse shoulder arthroplasty, Systematic review, Rotator cuff arthropathy, Irreparable rotator cuff tear, Patient reported outcome measures

## Abstract

**Background:**

Excellent short-term outcomes after reverse shoulder arthroplasty (RSA) have been reported, but longer term outcomes in the existing literature are sparse and vary widely. The purpose of this study is to systematically assess the existing literature to quantify functional outcomes and complication rates after RSA at a minimum of five years of follow-up.

**Methods:**

A Preferred Reporting Items for Systematic Reviews and Meta-analyses-compliant systematic literature search of the PubMed and Embase databases was undertaken. Studies reporting outcomes after primary RSA for nontrauma-related indications with a minimum of 5-year follow-up were included.

**Results:**

Overall, 20 studies satisfied all inclusion criteria. This represented 1591 shoulders in 1556 patients (32.1% males), with a mean age of 70.2 ± 5.0 years and mean follow-up of 8.8 years, or 106.2 ± 30.1 months (60-243). At final follow-up, the mean reported Constant Murley score was 62.1 ± 5.0 (49.0-83.0). The mean adjusted Constant Murley score was 83.5 ± 12.5 (58-111.9). The mean American Shoulder and Elbow Surgeons score was 81.8 ± 4.6, while the mean subjective shoulder value was 74.6 ± 6.4. Overall, 88% of patients rated their satisfaction as either good or very good. The range of active forward flexion, abduction, external, and internal rotation were respectively, 126° ± 13°, 106° ± 11°, 22° ± 11°, and 6° ± 2°. The overall rate of revision surgery was 4.9% (0%-45.5%). Regarding complications, the rate of prosthetic joint infection was 4.3% (0%-26.7%), shoulder dislocation was 3.7% (0%-20.4%), and acromial fracture was 2.0% (0%-8.8%). At final follow-up, 30.9% of shoulders had some degree of scapular notching.

**Conclusion:**

This systematic review shows that RSA results in high satisfaction rates, good clinical outcomes, as well as modest complication and revision rates at minimum 5-year follow-up.

Reverse shoulder arthroplasty (RSA) is a tool used in the treatment of a wide variety of degenerative and traumatic pathologies and as an option for salvage revision arthroplasty.^47^ Early prostheses were limited by a high rate of complications, including revision.^17^ In the past two decades, there has been a rapid proliferation in RSA innovation and in the volume of RSA operations being performed.^28^ Between 2011 and 2017, there was a 200% increase in the number of RSA performed in the United States, with an annual incidence of 20 per 100,000 adults, with similar increases seen worldwide.^23^^,^^34^^,^^36^^,^^39^^,^^49^ The incidence of RSA is rising at a far greater rate than that of other large joint arthroplasty, highlighting the growing importance of this procedure within the field of orthopedics.^57^ These trends may be partially explained by demographic changes, expanded surgical indications, and increased surgical familiarity with the procedure.^3^^,^^30^ However, given that modern RSA is a relatively recent surgical innovation, mid- to long-term outcomes in the published literature are sparse.^9^^,^^19^

RSA is known to result in excellent short-term outcomes, as has been shown in a recent large systematic review and meta-analysis with minimum follow-up of 2 years.^19^ In this review, RSA resulted in improved range of motion (ROM) in all planes and large clinically significant improvements in both Constant Murley (CM) score and American Shoulder and Elbow Surgeons (ASES) score, with a complication rate of 9.4% and revision rate of 2.6%.^19^ However, trials with longer follow-up (≥5 years) suggest that complication rates rise and function may decline with greater follow-up duration; this adds uncertainty regarding the long-term benefits from RSA.^5^^,^^7^^,^^13^^,^^48^ The purpose of this study is to systematically assess the existing literature to quantify functional outcomes and complication rates at a minimum of five years follow-up postprimary RSA. The hypothesis is that there will be a low revision rate, moderate complication rates, and good functional outcomes.

## Material and methods

### Study selection

Two independent reviewers (S.D. and E.H.) performed a literature search with respect to the Preferred Reporting Items for Systematic Reviews and Meta-analysis guidelines. This search was undertaken on December 31, 2022, and utilized the Pubmed and Embase databases. The search string employed was “reverse shoulder AND (arthroplast∗ OR replacement OR prosthesis)”. It was predetermined that no limits were to be applied to this search. Duplicate studies were removed manually. The two reviewers independently screened titles and abstracts, applying our exclusion criteria. Then both reviewers examined the full texts of remaining studies applying the inclusion criteria below. In cases of intraobserver disagreement, the senior author (M.M.) was predetermined to act as the final arbiter on inclusion.

### Eligibility criteria

Prior to the commencement of the search, criteria for inclusion and exclusion of trials were determined, along with a standardized data collection sheet. The inclusion criteria for this review included: [1] clinical studies reporting outcomes after primary RSA performed for nontraumatic indications with at least 5 years of follow-up; [2] published in the English language; and [3] published in a peer-reviewed journal. Exclusion criteria included: [1] all review articles; [2] cadaveric, in-vitro, and biomechanical studies; [3] abstracts, conference papers, and studies without an available full text; [4] studies of RSA performed for acute proximal humeral fracture, proximal humeral fracture sequalae, or as a revision arthroplasty; [5] studies of multiple treatments that did not adequately report on RSA as a subgroup; [6] studies including multiple different neck shaft angle (NSA) implants.

### Data extraction

Both reviewers independently collected data in our predetermined password-protected database on Microsoft Excel (Microsoft Corp., Redmond, WA, USA). Relevant data was extracted including: [1] year of publication and author; [2] study design; [3] level of evidence; [4] minimum and mean follow-up; [5] demographic data on trial participants; [6] clinical indication for RSA; [7] clinical and functional outcomes including patient-reported outcome measures; and [8] all relevant complications.

### Assessment of evidence

All included studies were assessed for their reported level of evidence using the Journal of Shoulder and Elbow Surgery criteria. The methodological quality of evidence was assessed using the Modified Coleman Methodology score (Supplementary Appendix S1). A score of <55 was considered to represent poor quality, 55-69 was considered fair quality, 70-84 was considered good quality and a score ≥85 was considered excellent quality.^35^ In cases of intraobserver disagreement, the senior author (M.M.) was predetermined to act as the final arbiter.

### Statistics

Quantitative statistical parameters were analyzed using Jamovi open-source statistical software (version 2.3.19; Jamovi Project, Tighes Hill, NSW, Australia). *P* values <.05 were considered statistically significant.

## Results

### Literature search

**Figure 1:**
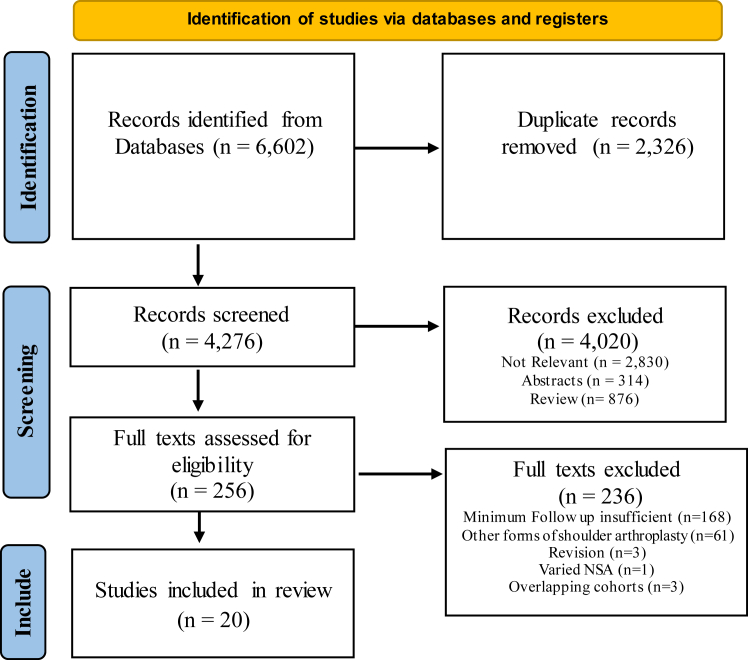
PRISMA flow diagram (Preferred Reporting Items for Systematic Reviews and Meta-Analyses). *NSA*, neck shaft angle.

The literature search yielded a total of 6602 studies. After manual removal of 2326 duplicate studies, the 4276 remaining studies were screened using our exclusion criteria. Thereafter, the inclusion criteria were used to judge the abstract and then full texts of the remaining 256 studies in order to evaluate eligibility. Overall, 20 clinical studies published between 2003 and 2022 were included in this review.^2^^,^^4^^,^^7^^,^^8^^,^^11^^,^^13^^,^^15^^,^^16^^,^^20^^,^^24^^,^^32^^,^^38^^,^^41^^,^^43^^,^^50^^,^^51^^,^^54^^,^^44^^,^^60^ There were three studies that reported a number of RSA performed for traumatic indications but reported results by indication; traumatic cases were excluded from the systematic review.^2^^,^^43^^,^^51^ The Preferred Reporting Items for Systematic Reviews and Meta-analysis flow chart representing this process is illustrated in Figure 1. The included studies constitute 1556 patients and 1,1591 shoulders, with 32.1% being male, a mean age of 70.2 ± 5.0 years, a mean body mass index of 29.5 ± 1.3 kg/m^2^, and a mean follow-up of 106.2 ± 30.1 months. There was one study that used a 135° NSA implant,^12^ 3 studies that used 145^o^ NSA implants,^43^^,^^50^^,^^52^ while the remainder used 155° NSA implants. Individual trial data is displayed in Table I.

**Table I:** Study characteristics and patient demographics.

Author	Year	LOE	MQOE	Shoulders	Patients	Male %	Age (y)	Follow-up (mo)	Indication	NSA
Bassens, et al^2^^,^∗	2019	3	48	66	66	28.4	69.4 ± 13	113 (97-135)	RCA, AVN, IA	155°
Bulhoff, et al^4^	2022	4	48	27	27	14.8	72.6 ± 5.4	127 (83-185)	OA, RCA	155°
Collin, et al^7^	2019	4	69	49	45	26.7	74.5 ± 6.3	92 (60-192)	OA	155°
Collotte, et al^8^	2021	3	45	97	97	23.7	74 ±7.5	101 (60-NR)	OA, IRCT, RCA, RA, IA	155°
Cuff, et al^11^	2022	3	44	93	93	73.2	69 ± 7	94 (74-132)	OA	135°
Ek, et al^13^	2013	4	52	40	35	68.6	60 ± 9	93 (60-171)	IRCT	155°
Ernstbrunner, et al^14^	2017	4	42	23	20	55	57 ± 3	140 (96-228)	IRCT	155°
Favard, et al^16^	2011	4	58	148	138	NR	73 ± 12.5	90 (85-107)	OA, IRCT, RCA	155°
Gerber, et al^20^	2018	4	37	22	22	33.3	71 ± 8.5	193 (180-228)	IRCT	155°
Imam, et al^24^	2020	3	56	100	94	34.8	74.5 ±8	NR (60-NR)	OA, RCA	155°
Lévigne, et al^32^	2021	3	64	40	35	14.3	69 ± 10	92 (60-147)	RA	155°
Mazaleyrat, et al^38^	2021	3	58	132	132	22	74.9 ± 5.4	108 (60-247.2)	OA, IRCT, RCA	155°
Nielsen, et al^41^	2022	3	50	78	78	33.3	71.2 ± NR	127 (120-160)	RCA	155°
Nourissat, et al^42^^,^∗	2022	4	53	38	38	16.7	73.8 ± 7.7	80 (73-93)	OA, IRCT, RCA	145°
Schoch, et al^50^	2021	4	46	165	165	32.1	71 ± 7.8	84 (60-132)	OA, IRCT, RCA	145°
Sebastia-Forcada, et al^51^^,^∗	2020	2	53	64	64	32.8	70.6 ± 6.1	101 (60-132)	RCA	155°
Simovitch, et al^52^	2019	3	58	324	324	32.4	72.0 ± 7.0	75 (60-132)	OA, IRCT, RCA	145°
Spiry, et al^54^	2021	3	58	45	45	NR	74.6 ± 5.3	145 (91-243)	OA, IRCT, RCA	155°
van Ochten, et al^44^	2019	3	60	27	27	3.7	73 ± 11	110 (60-NR)	OA, RCA, RA	155°
Woodruff, et al^60^	2003	4	66	13	11	NR	64 ± 7.3	87 (60-110)	RA	155°

*LOE*, level of evidence; *MQOE*, methodological quality of evidence; *NSA*, neck shaft angle; *NR*, not reported; *AVN*, avascular necrosis; *IA*, instability arthroplasty; *RSA*, reverse shoulder arthroplasty; *OA*, osteoarthritis; *RCA*, rotator cuff arthropathy; *IRCT*, irreparable rotator cuff tear; *RA*, rheumatoid arthritis.

∗ Indicates traumatic indications were excluded.

### Assessment of evidence

Of the 20 included studies, there was 1 level II study, 10 level III studies, and 9 level IV studies. The mean methodological quality of evidence, assessed by the Modified Coleman Methodology Score, was 53.3 ± 8.2, representing overall poor methodological quality with a range of 37-69.

### Functional outcomes

The most commonly reported functional outcome score was the CM score, which was reported in 16 trials of 1188 shoulders. The mean score was 62.1 ± 5.0 representing a mean preoperative improvement of 34.2 after at least 5 years of follow-up. There was improvement across all four domains of the CM score. The CM score adjusted for sex and age was reported in 12 trials of 552 shoulders with a mean result of 83.5 ± 12.5, and a mean improvement of 48.7. The ASES score was reported in 4 trials of 620 shoulders with a mean result of 81.8 ± 4.6 and improvement of 44.5. Patient satisfaction was reported in 5 studies of 205 shoulders, with 88% of participants either satisfied or very satisfied. Six studies of 278 shoulders reported subjective shoulder value with a mean result of 74.6 ± 6.4 and mean improvement of 51.1. There was a clinical improvement in all reported scores, as outlined in Table II.

**Table II:** Clinical outcomes at final follow-up.

Outcome	Studies (N)	Shoulders (N)	Mean score	Range	Mean improvement
CM	16	1188	62.1 ± 5.0	52.3-68.6	+34.2
Pain	8	451	12.9 ± 0.9	12.0-15.0	+8.2
Activity	5	366	16.5 ± 0.9	15.8-18.0	+9.3
Mobility	5	366	24.7 ± 4.1	20.0-31	+11.6
Strength	8	451	6.4 ± 1.8	4.0-9.3	+5.2
CM adjusted	12	552	83.5 ± 12.5	60-105	+48.7
ASES	4	620	81.1 ± 4.6	78.1-87.9	+44.5
SSV	6	278	74.6 ± 6.4	66-85	+51.1
SST	4	609	8.7 ± 1.1	7.1-9.4	+5.6
UCLA	3	553	28.2 ± 1.3	26.6-29.1	+15.0
DASH	1	64	26.1	/	/
SPADI	1	324	29.9	/	/
WOOS%	1	78	71.7	/	/
% Satisfaction	2	130	77% ± 10%	70-84%	/
Satisfied/Very Satisfied	5	205	88 ± 11%	72-96%	/
Forward flexion	14	1164	126° ± 13°	101°-149°	+50°
Abduction	8	692	106° ± 9°	86°-113°	+42°
External Rotation	13	1088	22° ± 11°	6°-41°	+8°
Internal Rotation	6	739	6° ± 2°	3°-8°	+3°

*N*, Number; *CM*, Constant Morley; *ASES*, American Shoulder & Elbow Surgeons; *SSV*, subjective shoulder value; *SST*, simple shoulder test; *UCLA*, University of California Los Angeles; *DASH*, disabilities of the arm, shoulder and hand; *SPADI*, Shoulder Pain and Disability Index; *WOOS*, Western Ontario Osteoarthritis of the Shoulder % of max score.

Forward flexion was the most common ROM metric reported, with 14 studies of 1164 shoulders returning a mean result of 126° ± 13°, an improvement of 50° from preoperative measurement. There was a similar large improvement of 42° of abduction, to a total of 106° ± 11°. However, there was small mean improvement in external and internal rotation of 8° and 3°, respectively, to a total of 22° ± 11° and 6° ± 2°. When assessing mean ROM across prosthesis NSA subgroups, there were 582 shoulders in the 155° NSA group, 489 in the 145° group, and 93 in the 135° group. Geater external rotation of 41° was reported in the 135° group, compared to 33° ± 0° in 145° group and 17° ± 8° in the 155° group. While flexion, abduction, and internal rotation were all similar between groups, this is displayed in Table III.

**Table III:** Shoulder range of motion grouped by prosthetic neck shaft angle.

	155° NSA			145° NSA			135° NSA		
	N	Mean	Improvement	N	Mean	Improvement	N	Mean	Improvement
Forward flexion	582	124° ± 13°	+50°	489	129° ± 1°	+39°	93	149°	+24°
Abduction	203	104° ± 10°	+43°	489	111° ± 2°	+35°	/	/	/
External rotation	555	17° ± 8°	+8°	489	33° ± 0°	+11°	93	41°	+18°
Internal rotation	250	6° ± 2°	+3°	489	5° ± 0°	+1°	/	/	/

*NSA*, neck shaft angle; *N*, number.

### Radiological outcomes

Scapular notching was reported in 11 studies of 914 shoulders, all of which used the Sirveaux grade to classify notching as grade 1-4. The overall rate of notching in 914 shoulders was 30.9% at a mean follow-up of 111 ± 37 months. The rate of grade 1-2 notching was 20.6%, and the rate of grade 3-4 notching was 10.4%. The majority of data (64.6%) came from 155° NSA implant trials, with the remainder using 145° NSA prosthetics. Scapular notching grouped by implant NSA can be seen in Table IV. The 145° NSA implant appears to be associated with lower rates of notching over longer term follow-up.

**Table IV:** Scapular notching rate overall and by neck shaft angle.

Prosthetic	Total	155° NSA	145° NSA
Studies (N)	N =11	N = 10	N =1
Follow-up (mean ± SD)	111 ± 37	115 ± 37	75

Scapular Notching Sirveaux Grade	Total	%	Total	%	Total	%
Grades 1 or 2	188/914	20.6	149/590	25.3	39/324	12.0
Grades 3 or 4	95/914	10.4	87/590	14.7	8/324	2.5
Grade 1-4	283/914	30.9	236/590	40	47/324	14.5

*NSA*, neck shaft angle; *N*, number; *SD*, standard deviation.

When assessing all trials reporting notching, 27% (3/11) reported a significant difference for worse functional outcomes after RSA in the notched group compared to no notching group. 46% of trials (5/11) found no significant difference between the two groups, while 27% (3/46) did not report a comparison between the groups. When comparing grade 1-2 notching with grade 3-4 notching, 18% of trials (2/11) found significantly worse functional outcomes in the more severe notching group, 27% found no significant difference (3/11), and it was unreported in 55% of trials (6/11). When assessing the relationship between notching and revision, 9% (1/11) reported a significant relationship between grade 4 notching and glenoid implant failure.^54^ While assessing only studies with 10 or more years of follow-up, there were 117 shoulder all with 155° NSA implants with an overall rate of notching equal to 71.8%, with 42.7% having grade 1-2 and 29.1% having grade 3-4 notching.

There was limited reporting on tuberosity reabsorption rates. Collin et al reported greater tuberosity (GT) reabsorption partially in 8% and totally in 4% of shoulders, with lesser tuberosity reabsorption in 16% partially and 6% totally.^7^ Levigne et al reported GT reabsorption partially in 7.5% of shoulders, and 2.5% had total reabsorption.^32^ While Mazaleyrat et al reported GT reabsorption in 47% of shoulders without defining the degree of absorption.^38^

### Complications and revisions

Overall, 8 studies reported on the following complications. The rate of glenoid periprosthetic fracture in 954 shoulders was 0.8%, with reported rates ranging from 0% to 4.5%. This data is displayed in Table V. Humeral periprosthetic fractures in 954 shoulders occurred at a rate of 2.4%, with reported rates from 0% to 4.5%. The rate of acromial fractures ranged from 0% to 6.7%, and in 808 shoulders, the overall rate was 2.0%.

**Table V:** Complications and revisions.

Outcome	Studies (N)	Follow-up	Total	%
Complications				
Periprosthetic infection	9	106 ± 34	40/935	4.3
Glenoid periprosthetic fracture	8	101 ± 39	8/954	0.8
Humeral periprosthetic fracture	8	101 ± 39	23/954	2.4
Prosthetic disassembly	5	117 ± 21	5/384	1.3
Acromial fracture	8	90 ± 18	16/808	2.0
Nerve palsy	8	100 ± 19	18/597	3.0
Aseptic loosening	14	115 ± 30	40/1147	2.4
Dislocations	14	106 ± 34	48/1290	3.7
Revision				
Revision arthroplasty	18	106 ± 31	80/1617	4.9

There were 9 studies which reported on periprosthetic infection (PJI) with rates ranging from 0% to 27%. Among 935 shoulders, the rate of PJI was 4.3%. While 5 studies reported on prosthetic disassembly with reported rates ranging from 0.8% to 4.4%, overall among 384 shoulders, the prosthetic disassembly rate was 1.3%. The reported rate of aseptic loosening ranged from 0% to 18.2%, and among 1147 shoulders in 14 studies, the overall rate was 2.4%. The reported rate of shoulder dislocation from 14 studies ranged from 0% to 17.5%, and among 1290 shoulders, the rate of dislocation was 3.7%.

There were 8 studies that reported on the nerve injuries with rates ranging from 0% to 7.5%. Overall, the reported rate of nerve injuries was 3.0% among 597 shoulders; no reported nerve injuries were reoperated on. Thirty-nine percent of injuries were to the axillary nerve, 39% to the ulnar nerve, 11% to the radial, and 11% to unspecified brachial plexus injuries.

Overall, 18 studies with a mean follow-up of 106 ± 31 months reported upon revision surgeries, with rates ranging from 1% to 45%. The overall revision rate for the 1617 patients was 4.9%. When this was limited to studies with greater than 10 years of follow-up; there were 4 studies with a mean follow-up of 147 ± 31 months; the revision rate for these 150 patients was 14%.

## Discussion

The most important finding of this systematic review of 1591 shoulders is that primary atraumatic RSA results in good functional outcome scores and ROM at minimum 5-year follow-up, with high patient reported satisfaction rates. Importantly, patients and surgeons should be reassured by the modest reported revision and complication rates.

There is a lack of reliable data on the minimal clinically important difference [MCID] for functional outcomes after shoulder arthroplasty. The longest follow-up for a study reporting MCIDs after primary nontraumatic RSA is 6 years.^53^ In this systematic review, mean functional outcome scores are reported that are significantly greater (*P* < .0001 for all, T-test) than the published MCID for RSA, with an ASES score (44.5 vs. 21.7), CM (34.2 vs. 11.6), University of California Los Angeles shoulder score (15.0 vs. 9.7) and Simple Shoulder Test (SST) (5.6 vs. 3.3). These outcomes also exceed the MCID which have been reported with 1-2 years follow-up post-RSA.^19^^,^^56^^,^^61^ The ROM outcomes are similarly positive with significantly greater improvements compared to the MCID in forward flexion (50° vs. 19.7°) and abduction (42° vs. 18.2°) (*P* < .0001), while achieving the MCID for external rotation (8° vs. 7.3°) (*P* = .148).^53^ These results indicate that RSA offers a durable long-term benefit that is clinically relevant. This mirrors the findings of the smaller systematic review by Ernstbrunner et al with ≥5-year follow-up including 365 shoulders at a mean follow-up of 9.5 years, which reported equivalent patient-reported outcome measure and ROM data to current review and also demonstrated a lack of substantial functional decline over prolonged follow-up.^14^ Galvin et al reported in their systematic review of 5824 shoulders with a follow-up ≥2 outcomes that are equivalent to the present study, with mean improvements in flexion of +56°, abduction +50°, external rotation +14°, ASES score +42, and CM score +37. The absolute values and postoperative improvements they report were greater although the differences are of limited clinical relevance and universally less than the previously stated MCIDs.^19^ The improvements in function seen at two years postoperatively, although liable to decline appear to be robust over longer follow-up, with patients having good function and high rates of reported satisfaction. Encouragingly, Mathew et al have reported from an institutional registry of 1899 shoulders that 5-year outcomes for RSA improved year-on-year from 2008-2018 when looking at each calendar year as a cohort. Patients undergoing RSA today can reasonably be expected to match or outperform the figures reported here.^37^

It has previously been reported by large systematic review that RSA confers significant clinical improvements regardless of the indication.^9^ However, in systematic reviews by Coscia et al and Paras et al, worse functional outcomes and higher complications rates for traumatic indications are reported compared to RSA performed for elective conditions.^9^^,^^45^ While revision RSA is also associated with poorer outcomes and higher rates of complications than primary RSA.^10^^,^^22^^,^^58^ Hence, primary atraumatic indications were selected for this systematic review. The figures reported for functional outcomes in Table I show a relatively narrow range of data indicating a consistent treatment effect across different indications. The Nourissat et al study was the only study in this systematic review to compare functional outcomes between cuff tear arthropathy, osteoarthritis, and irreparable rotator cuff tear with no statistically or clinically significant difference between groups postoperatively.^42^

Comparison of outcomes based on NSA in this review should be undertaken with caution due to baseline differences in the groups, varied durations of follow-up, and the relatively small amount of data available for the subgroups. It has previously been reported in biomechanical and clinical studies that implants with NSA <155° have greater range of motion particularly in external and internal rotation, and lower rates of notching.^33^^,^^40^^,^^46^^,^^59^^,^^62^ Table III highlights that all groups experienced substantial improvements in range of motion. The <155° implant groups obtained clinically relevant greater range in external rotation (41° for 135° NSA, 33° for 145° NSA, and 17° for 155° NSA). While they also obtained greater final flexion and abduction, although these differences were less significant (Table III). The <155° implant groups show greater final ROM but a smaller mean improvement. This may be explained by a ceiling effect on possible improvement and/or baseline differences between groups. Table IV explores the rate of scapular notching between NSA subgroups, showing a trend of lower rates of notching and severe notching in the 145° NSA group. Statistical testing between the 155° and 145° groups was not performed as the authors’ felt it was not appropriate due to the differences between the groups. While acknowledging the aforementioned limitations, this long-term follow-up data indicates possible benefit with respect to notching and humeral rotation in <155° NSA implants. Further prospective trials will be required to explore these findings.

There is limited and contradictory published data reporting on the clinical relevance of scapular notching.^14^^,^^18^^,^^29^^,^^55^ A meta-analysis with a minimum 2-year follow-up and 1532 patients assessed the effect of scapular notching on clinical outcomes. They found notching resulted in statistically significantly lower scores for CM, ASES, forward flexion abduction but not for external rotation in the notched group.^25^ This analysis is limited by the quality of the mainly retrospective studies included and the fact that they do not delineate data based upon the grade of notching. Adverse outcomes may have been driven primarily by cases of advanced notching. The current review shows that the prevalence of notching increases over time (30.9% vs. 71.8% at mean follow-up of 9.3 years vs. 12.6 years), and the prevalence of more severe notching (grade 3-4) also increases over the same time periods (10.4%-29.1%). Almost 1 in 3 patients in this review had scapular notching at final follow-up, yet excellent functional outcomes are reported. This highlights that the presence of notching is not a guarantee of impaired outcomes; it is a multifactorial process and not purely related to the presence or degree of scapular notching. Of the included studies, 27% reported a significant difference in functional outcomes between notched and un-notched groups, and just 18% reported a significant difference between mild and major grades of notching. Hence, a majority of trials either did not find a significant difference or did not report a comparison. There is a need for quality prospective controlled longitudinal studies to isolate the clinical effects of the prevalence and severity of notching.^6^^,^^27^^,^^33^

The reporting of complications was heterogeneous and inconsistent throughout the included studies. Patients who experienced complications may be more likely to take part in prolonged follow-up, while others will have failed to complete the 5-year follow-up as a result of their complications, leading to a survivorship bias. It is also known that complications are inherently linked to the population and indication for RSA.^14^ Due to the variations in reporting, the authors felt calculating an overall complication rate would not be appropriate. Although unsurprisingly, there does appear to be a trend of increased complications associated with longer term follow-up.^19^ The reported rate of revision from 1617 shoulders was widely heterogeneous (1%-45%), which is not unexpected while allowing for variations in prosthesis, sample size, and duration of follow-up. The overall rate was 4.9%, and when restricting for ≥10 years of follow-up, it was 14% in 150 shoulders. The final follow-up figures are line with revision rates of 3 and 5% reported in recent registries with long-term follow-up.^21^^,^^26^ The 10-year revision rate is reason for some concern, although this should be tempered by the comparatively small sample size, which was 1/10 of the 5-year follow-up group. The Danish and Nordic shoulder registries included 1699 and 1904 shoulders at 10 years and reported revision rates of 8.5% and 7.9%, results that are likely to lie closer to the true rate.^1^^,^^31^ The registry studies report PJI, instability, and prosthetic loosening as the primary causes of revision. These complications were the major driver of revision in this review, although overall they were rare events.

### Limitations

Limitations of this review include those inherent to the included studies. The vast majority of included trials are at levels III and IV, highlighting the dearth of high-quality evidence with long-term follow-up. Another limitation is that 8/20 studies (40%) excluded those who underwent revision from their functional outcome scores. It is important that surgeons are cognizant of the potential for poor functional outcomes after complications. Another limitation was the heterogeneous and inconsistent reporting of complications across trials. The varied surgical indications present in this review may be considered a limitation, although this is representative of the primary atraumatic indications for RSA and of the published literature.

## Conclusion

This systematic review shows that RSA results in high satisfaction rates, good clinical outcomes, as well as modest complication and revision rates at minimum 5-year follow-up.

## Disclaimers:

Funding: No funding was disclosed by the authors.

Conflicts of interest: The authors, their immediate families, and any research foundation with which they are affiliated have not received any financial payments or other benefits from any commercial entity related to the subject of this article.
