# Supplementary material for: Midterm outcomes of primary reverse shoulder arthroplasty: a systematic review of studies with minimum 5-year follow-up
Source: JSES Rev Rep Tech. 2023 Oct 3;4(1):1–7. doi: 10.1016/j.xrrt.2023.09.003 (PMC10840579; doi:10.1016/j.xrrt.2023.09.003)
Supplement: Supplementary Appendix S1 [file mmc1.docx]

﻿

**Modified Coleman Methodology Score**

**Part 1: One score for each of the sections:**

1. Number of patients:

| a. <30 | 0 |
| --- | --- |
| b. 30-50 | 4 |
| C. 51-10 | 7 |
| d. >100 | 10 |

2. Mean follow-up

| a.<12 months | 0 |
| --- | --- |
| b.12-36 months | 4 |
| C.37-60 months | 7 |
| d. >61 months | 10 |

3. Surgical approach

| a. Different approaches and outcome not reported separately | 0 |
| --- | --- |
| b. Different approaches and outcome reported separately | 7 |
| c. Single approach | 10 |

4. Type of study

| a. Retrospective cohort study | 0 |
| --- | --- |
| b. Prospective cohort study | 10 |
| c. Randomized controlled trial | 15 |

5. Description of diagnosis

| a. Described without percentage specified | 0 |
| --- | --- |
| b. Described with percentage specified | 5 |

6. Description of surgical technique

| a. Not stated/unclear – Inadequate | 0 |
| --- | --- |
| b. Only stated - Fair | 5 |
| c. Stated with details – Adequate | 10 |

7. Description of postoperative rehabilitation

| a. Described | 5 |
| --- | --- |
| b. Not described | 0 |

**Part 2: Scores can be assigned for each option of every section**

1. Outcome criteria

| a. Outcome measures clearly specified | 2 |
| --- | --- |
| b. Timing of outcome measures clear | 2 |
| c. Outcome measures with reported reliability | 3 |
| d. General health measure included | 3 |

2. Outcome assessment

| a. Participants recruited | 5 |
| --- | --- |
| b. Investigator independent of surgeon | 4 |
| c. Written assessment | 3 |
| d. Assessment completed by patients | 3 |

3. Description of participants selection process

| a. Selection criteria reported and unbiased | 5 |
| --- | --- |
| b. Recruitment rate reported (>90%) | 5 |
| c. Recruitment rate reported (<90%) | 0 |

| **TOTAL** |  |
| --- | --- |

<55 = poor quality,

55-69 = fair quality,

70-84 = good quality

≥85 = excellent quality
